# Incidence of contrast-induced acute kidney injury (CI-AKI) in high-risk oncology patients undergoing contrast-enhanced CT with a reduced dose of the iso-osmolar iodinated contrast medium iodixanol

**DOI:** 10.1371/journal.pone.0233433

**Published:** 2020-05-21

**Authors:** Sebastian Werner, Christian Bez, Clemens Hinterleitner, Marius Horger

**Affiliations:** 1 Department of Diagnostic and Interventional Radiology, University Hospital Tübingen, Tübingen, Germany; 2 Department of Internal Medicine II–Haematology, Oncology, Clinical Immunology and Rheumatology, University Hospital Tübingen, Tübingen, Germany; A.C. Camargo Cancer Center, BRAZIL

## Abstract

**Objectives:**

To determine the incidence of post-contrast acute kidney injury (PC-AKI) and presumed contrast-induced acute kidney injury (CI-AKI) following contrast-enhanced CT (CECT) with intravenous application of a reduced dose of the iso-osmolar contrast agent iodixanol in cancer patients with chronic kidney disease.

**Methods:**

198 oncology patients with a baseline estimated glomerular filtration rate (eGFR) <60ml/min/1.73m^2^ undergoing a total of 237 CECTs using a reduced dose of 60ml iodixanol were retrospectively analyzed. Statistical analysis was performed for the entire cohort and subgroups. The effect of additional risk factors on the occurrence of PC-AKI was evaluated.

**Results:**

The overall PC-AKI incidence was 6.3%. Excluding patients with concurrent medical conditions known to directly and independently impact kidney function and patients with AKI preceding the CT-scan resulted in a presumed CI-AKI incidence of 3.8%. No permanent post-contrast worsening of renal function and no AKI treatment were required. Subgroups considering baseline eGFR yielded PC-AKI incidences of 4.6% (eGFR 45-60ml/min/1.73m^2^, n = 130), 7.4% (eGFR 30-45ml/min/1.73m^2^, n = 95) and 16.7% (eGFR <30ml/min/1.73m^2^, n = 12). Additional patient related risk factors did not show any significant effect on the occurrence of PC-AKI.

**Conclusions:**

Low incidences of PC-AKI/CI-AKI suggest that a reduced dose of an iso-osmolar contrast agent is safe in high-risk oncological patients with impaired renal function.

## Introduction

Contrast-induced acute kidney injury (CI-AKI) is a potential complication of intravascular administration of iodinated contrast agents and is associated with increased morbidity and mortality [1–4]. It is defined as a sudden deterioration of renal function that is caused by the administration of the contrast agent. The diagnosis of CI-AKI requires the absence of any other concurrent medical condition that could also be the cause of the worsening of kidney function [5]. CI-AKI is considered a subentity of post-contrast acute kidney injury (PC-AKI) which is defined as a sudden deterioration of renal function following contrast administration regardless of whether the contrast medium was the cause of the deterioration [6, 7].

In clinical practice it is difficult to clearly identify cases of PC-AKI that have been caused by the contrast agent and permit the diagnosis of CI-AKI because PC-AKI can also be caused by a variety of other coincident nephrotoxic events or simply reflect spontaneous fluctuations in serum creatinine (SCr) or estimated glomerular filtration rate (eGFR) [6, 7].

The incidence of CI-AKI has been reported to be variable and to depend mainly on the contrast medium osmolality and applied volume [5, 8–10]. Reports from the 1980s and 1990s indicated significantly higher rates of CI-AKI [11, 12]. This was presumably caused by higher volumes of administered contrast agent made necessary by significantly longer examination times in the pre-helical-CT era. Especially for larger body coverage (e.g. tumor staging), repeated contrast injections were needed, increasing consecutively the volume of applied contrast agent. Additionally, the preponderant use of high-osmolar contrast agents is presumed to have been responsible for higher reported rates of PC-AKI.

More recently, published data on this topic have demonstrated lower rates of PC-AKI with an overall incidence around 5.0% to 6.4% [1, 13]. The major challenge in assessing the true incidence of CI-AKI is the frequent coexistence of additional risk factors, particularly in inpatients [14]. The lack of propensity matching leads to biased data which include patients already at risk for acute kidney injury (AKI) [14, 15].

Cancer patients are at an increased risk of experiencing CI-AKI [16–18]. Many of these patients require frequent contrast-enhanced CT (CECT) examinations, each with a potential nephrotoxic effect. Furthermore, a bidirectional relationship exists between cancer and kidney function which varies among the different cancer types but is related to cancer-accompanying nephrotoxic drugs, dehydration, higher patient age and paraneoplastic renal disorders [19]. Oncological patients presenting with impaired kidney function (eGFR<60ml/min/1.73m^2^) are at even higher risk for CI-AKI [20, 21]. Moreover, the rate of CI-AKI has been reported to rise with an increasing number of risk factors [5, 13, 22, 23].

In knowledge of this risk constellation, different strategies aiming at risk stratification have been recommended [14, 24]. They consider the baseline eGFR as well as comorbidities or medical conditions potentially affecting renal function and recommend reduction of contrast volume and the use of iso-osmolar contrast media (IOCM) [9, 19].

Hence, the aim of this retrospective study was to assess the incidence of CI-AKI in a cohort of high-risk oncology patients with impaired renal function undergoing CECT with a reduced dose of the IOCM iodixanol.

## Materials and methods

### Patient characteristics

This retrospective single cancer center evaluation of clinical and laboratory data was approved by the local institutional review board (Clinical Ethics Committee at the University Hospital of Tübingen, registration number 161/2019BO2). The need for written informed consent was waived by the ethics committee due to the retrospective nature of the study. A total of 237 CECT-examinations in 198 oncology patients acquired between December 2016 and July 2018 were retrospectively identified by a database search for CECT using iodixanol in patients with impaired renal function. The average patient age was 70.0 years (range 33–95). 43% of exams were performed in women. Patients received on average 1.4 CECTs (range 1–4). Our standard protocol for all patients with an eGFR<60ml/min/1.73m^2^ requiring CECT prescribes a reduced dose of 60ml iodixanol. It was established years ago aiming at reducing the incidence of CI-AKI.

94 (39.7%) inpatients and 143 (60.3%) outpatients (including day-hospital patients) were examined. 198 (83.5%) CECT-examinations were elective whereas 39 (16.5%) were non-elective, i.e. patients were being referred to CECT for medical emergencies.

In 183 cases (77.2%) the underlying disease was a solid tumor, the most frequent diagnoses being lung cancer (n = 43) and colon cancer (n = 28). In 54 cases the underlying disease was a hematological malignancy, mantle cell lymphoma (n = 11) and diffuse large B-cell lymphoma (n = 10) being the most frequent.

The following eight additional risk factors for AKI were registered in all patients: status post renal transplantation, status post stem cell transplantation, diabetes mellitus, arterial hypertension, peripheral artery disease, congestive heart failure, concurrent nephrotoxic medication (excluding anti-cancer medication) and concurrent nephrotoxic anti-cancer medication. Registered non anti-cancer medication included Aminoglycosides, antiviral agents, Amphotericin B, Colistin, Polymixin B, Sulfadiazine, Quinolones, Vancomycin, NSAIDs, Selective COX-2 inhibitors, Phenacetin, Calcineurin inhibitors, Sirolimus, Everolimus, ACE inhibitors/ARBs/renin inhibitors, SGLT-2 inhibitors (canagloflozin, dapagliflozin), Methoxyflurane, Pamidronate, Zolendronate, Topiramate, Zonisamide, Orlistat, Statins, Mesalamine [25]. Registered anti-cancer medication included Cisplatin, Ifosfamide, Methotrexate, Pemetrexed, Ipilimumab, Gemcitabine, Mitomycin, Bevacizumab, Sunitinib, Sorafenib, Axitinib, Pazopanib, Cetuximab, Panitumumab and Imatinib [26].

Patients were grouped into three baseline eGFR subgroups which were based on the current KDIGO 2012 Clinical Practice Guideline for the Evaluation and Management of Chronic Kidney Disease [28]: baseline eGFR [ml/min/1.73m^2^] 45–60, 30–45 and <30, corresponding to KDIGO CKD GFR categories G3a, G3b and G4/5. All patients were in clinical, imaging and laboratory surveillance at our institution.

### Definition of PC-AKI and CI-AKI

PC-AKI was defined as an increase in SCr of ≥ 0.3 mg/dl or ≥ 50% above the baseline value within 72 hours after contrast administration. This definition is based on the current Clinical Practice Guideline for Acute Kidney Injury by “Kidney Disease: Improving Global Outcomes” (KDIGO) [27] and the older criteria of the Acute Kidney Injury Network (AKIN) [28]. It reflects the current recommendations by the ESUR CMSC [7] and the Committee on Drugs and Contrast Media of the American College of Radiology (ACR) [6].

SCr and eGFR (Chronic Kidney Disease Epidemiology Collaboration (CKD-EPI) equation) were determined at baseline (mean 1.5 days before the CT-scan; SD 2.4; range 0–11), and within 72 hours after contrast administration (mean 1.8 days after the CT-scan; SD 0.8; range 1–3). Normal values for SCr at our institution laboratory range from 0.5–0.8mg/dl for females to 0.6–1.1mg/dl for males.

Since the diagnosis of CI-AKI requires a deterioration of kidney function in the absence of any other cause [5] we further examined the medical records of PC-AKI cases to identify those in which such other known causes of AKI [27], i.e. sepsis, critical illness, circulatory shock, cardiac surgery, major non-cardiac surgery as well as post-renal ureteral obstruction were present. Additionally, we identified those who showed AKI immediately before CECT was performed. PC-AKI cases for which none of these conditions was identified were regarded as being caused by the contrast agent and are referred to as cases with *presumed CI-AKI*.

### CECT-examination protocol

The CT studies were performed with patients in supine position using 256-slice MDCT scanners (SOMATOM Definition Flash, Siemens Healthcare, Forchheim, Germany). The examination protocol included intravenous administration of 60ml IOCM (320mg iodine/ml Iodixanol [VISIPAQUE, GE Healthcare Buchler GmbH & Co. KG, Germany]) at a flow rate of 2ml/s via antecubital vein followed by a saline flush of 30ml NaCl at 2.5ml/s. Contrast material was administered by using a dual-head pump injector (CT motion XD 8000, Ulrich Medical, Ulm, Germany). Over 95% of patients received oral hydration 500ml before and after CECT whereas the rest received IV hydration with 500ml 0.9% NaCl before and after CECT.

### Statistical analysis

Statistical analysis was performed by using software (IBM SPSS Statistics Version 26). P<0.05 was indicative of statistical significance. Differences in PC-AKI rates between inpatients and outpatients as well as between elective and emergency cases were assessed via Fisher’s exact test for independent samples. Differences regarding SCr and eGFR values pre and post CECT were assessed using the Wilcoxon signed rank test for related samples. Logistic regression analysis was performed to determine effects of the baseline eGFR value and baseline eGFR subgroup as well as of the additional risk factors on the probability of the occurrence of PC-AKI.

## Results

### Incidence of PC-AKI and presumed CI-AKI with subgroup analysis

Rates of PC-AKI and presumed CI-AKI regarding the entire cohort and baseline eGFR subgroups are presented in Table 1.

**Table 1:** Incidence of PC-AKI and presumed CI-AKI in entire cohort and baseline eGFR subgroups.

	PC-AKI	Presumed CI-AKI
Entire cohort (n = 237)	6.3% (n = 15)	3.8% (n = 9)
Baseline eGFR 45–60 (n = 130)	4.6% (n = 6)	3.8% (n = 5)
Baseline eGFR 30–45 (n = 95)	7.4% (n = 7)	4.2% (n = 4)
Baseline eGFR <30 (n = 12)	16.7% (n = 2)	0.0% (n = 0)

eGFR [ml/min/1.73m^2^]

To investigate wether the baseline eGFR had any effect on the probability of the occurrence of PC-AKI we performed a logistic regression analysis, which showed that both the model as a whole (chi-square(1) = 0.568, p = 0.451, n = 237) and the individual coefficient (odds ratio 0.979, lower 95% CI 0.927, upper 95% CI 1.034, p = 0.445, n = 237) were not significant. Furthermore we were not able to show a significant correlation between the baseline eGFR subgroup and the occurrence of PC-AKI using Fisher’s exact test (p = 0.172).

When comparing elective (n = 198) and emergency cases (n = 39) the PC-AKI rates were 6.1% (n = 12) and 7.7% (n = 3). The respective CI-AKI rates were 3.5% (n = 7) and 5.1% (n = 2). According to Fisher’s exact test there were no significant differences between the two groups (p = 0.72 and 0.645, respectively). When comparing inpatients (n = 94) and outpatients (n = 143) the PC-AKI rates were 9.6% (n = 9) and 4.2% (n = 6). The respective CI-AKI rates were 4.3% (n = 4) and 3.5% (n = 5). There were no significant differences between these two groups either (p = 0.109 and 0.743, respectively).

A detailed medical chart review of patients with PC-AKI showed that none experienced permanent post-contrast worsening of renal function.

### SCr and eGFR pre and post CECT

The median SCr and eGFR for the entire cohort and baseline eGFR subgroups are depicted in Table 2. The respective absolute and relative changes of these values between pre and post CECT are shown in Table 3.

**Table 2:** Serum creatinine and eGFR at baseline and follow-up for entire cohort and baseline eGFR subgroups.

		Baseline		Follow-Up		
		SCr	eGFR	SCr	eGFR	p-value
Entire cohort (n = 237)	Median	1.40	46.50	1.37	46.38	0.108 (SCr), **0.019** (eGFR)
	IQR	0.35	12.75	0.44	16.36	
Baseline eGFR 45–60 (n = 130)	Median	1.30	52.23	1.20	52.25	0.569 (SCr), 0.103 (eGFR)
	IQR	0.30	7.19	0.30	12.72	
Baseline eGFR 30–45 (n = 95))	Median	1.50	39.97	1.50	39.95	0.117 (SCr), **0.033** (eGFR)
	IQR	0.30	5.93	0.4	10.47	
Baseline eGFR <30 (n = 12)	Median	2.35	26.32	2.15	24.95	0.373 (SCr), 0.328 (eGFR)
	IQR	0.75	6.63	1.18	14.38	

SCr: serum creatinine [mg/dl]

eGFR: estimated glomerular filtration rate [ml/min/1.73m^2^]

Bold p-values indicate statistical significance

**Table 3:** Absolute and relative difference in serum creatinine and eGFR between baseline and follow-up for entire cohort and baseline eGFR subgroups.

	sCr absolute difference		sCr relative difference		eGFR absolute difference		eGFR relative difference	
	Median	IQR	Median	IQR	Median	IQR	Median	IQR
Entire cohort (n = 237)	0.00	0.21	0.00	16.52	0.00	9.42	0.00	19.67
Baseline eGFR 45–60 (n = 130)	0.00	0.20	0.00	17.48	0.00	11.23	0.00	21.43
Baseline eGFR 30–45 (n = 95)	-0.06	0.21	-4.67	15.00	2.04	6.68	6.40	17.77
Baseline eGFR <30 (n = 12)	-0.05	0.61	-1.79	27.24	0.47	10.37	2.24	36.98

SCr: serum creatinine [mg/dl]

eGFR: estimated glomerular filtration rate [ml/min/1.73m^2^]

IQR: interquartile range

### Evaluation of additional risk factors

The number of additional risk factors in the cohort was: 0 (1.1%, n = 1), 1 (26.3%, n = 25), 2 (31.6%, n = 30), 3 (28.4%, n = 27), 4 11.6%, n = 11), 5 (1.1%, n = 1). The most prevalent risk factors were nephrotoxic medication in 88.6%, hypertension in 57.0% and concurrent nephrotoxic anti-cancer medication in 46.8%. These were followed by Diabetes mellitus (27.0), congestive heart failure (12.2%), status post stem cell transplantation (6.8%), peripheral artery disease (3.8%) and status post kidney transplantation (1.3%).

Logistic regression analysis was performed to investigate wether any of the additional risk factors had a significant effect on the probability of the occurrence of PC-AKI. Results showed that both the model as a whole (chi-square(8) = 10.592, p = 0.226, n = 237) and the individual coefficients of the variables were not significant.

## Discussion

The incidence of PC-AKI in our retrospective analysis of cancer patients with impaired renal function (corresponding to CKD GFR categories G3-G5) and high-risk for AKI who underwent CECT with a reduced-dose of iodixanol was 6.3%. Excluding PC-AKI cases in which other known causes of AKI [27] were present, as well as cases of pre-CECT AKI, resulted in a presumed CI-AKI incidence of 3.8%. Notably, on average there was no significant worsening of SCr and eGFR values between baseline and post contrast follow-up in any of the baseline eGFR subgroups (45–60 vs. 30–45 vs. <30 ml/min/1.73m^2^).

Earlier meta-analyses evaluating the overall risk for CI-AKI reported varying results, but recent data have shown the risk for deterioration of renal function to be around 5.0% to 6.4% in the general population [1, 13]. However, in patients with chronic kidney disease (CKD) the incidence of CI-AKI exceeds these rates with percentages being reported between 8.8% to 20% for low-osmolar contrast agents (LOCM) [1]. Moreover, the incidence of CI-AKI is expected to increase with every additional risk factor (e.g. diabetes mellitus, older age, congestive heart failure, sepsis, etc.) [5]. Oncology patients are at even greater risk for CI-AKI with incidences reported up to 20% [20]. In a large comparable cohort of cancer patients with known CKD undergoing contrast-enhanced CT, Ng et al. reported a risk of acute renal events of up to 10.5%, increasing with the stage of CKD [18]. The observed PC-AKI rates in our analysis are in the range of those reported for PC-AKI in the general population. This is in line with the findings by McDonald et al. showing that IV administration of the IOCM iodixanol represents no independent risk factor for AKI, dialysis, or increased mortality [29]. None of our cancer patients experiencing presumed CI-AKI showed persistent deterioration of renal function as compared to baseline. The risk of AKI in cancer patients has variable causes including the so-called kidney-cancer connection which has been statistically demonstrated to be significant in many tumor entities (e.g. renal cell carcinoma, hepatocellular carcinoma, myeloma, leukemia and lymphoma), paraneoplastic disorders (glomerular and tubular diseases) and in particular the nephrotoxic anti-tumor drugs [30]. Hence, CI-AKI in cancer patients is a multifactorial syndrome and due to the hypothetically limitless numbers of patient, disease and treatment characteristics is also difficult to trace back to a single trigger. In a large cohort of Danish inpatients, Christiansen et al. found a 1-year risk of AKI of 17.5% and 5-year risk of 27% with 5.1% of patients requiring dialysis within 1 year [16]. Similarly, Launay-Vaucher et al. found a high (57.2%) prevalence of renal impairment in a study of nearly 5,000 patients with solid tumors [17]. In order to reduce the rate of CI-AKI, eGFR-based risk stratification has been recommended knowing that values <45mL/min/1.73^2^ are at higher risk for AKI [31, 32]. However, most study reports evaluating the incidence of CI-AKI focused on low-osmolar contrast agents [11, 33]. Recent data support the use of iso-osmolar contrast media to prevent CI-AKI both for intravenous and intraarterial administration, particularly in the setting of CKD [34–37]. In a randomized clinical trial performed in cancer patients, Terrenato et al. reported a more favorable safety profile of Iodixanol vs. Iopromide [37]. Similar to our own results, the authors did not report any case of permanent post-contrast worsening of kidney function. Mehran et al. emphasized the increased risk of CI-AKI in patients with higher baseline creatinine values (CKD stages) with up to 62% CI-AKI after angiographic intervention for SCr levels above 2mg/dl [5]. Our own data also show an increase in PC-AKI incidence with decreasing levels of baseline eGFR. The incidence of PC-AKI in CKD GFR categories G4/5 more than doubled compared to the G3 subgroup (7.4% vs. 16.7%). Nevertheless, we found no statistically significant association between baseline eGFR subgroups and PC-AKI rates, possibly due to a relatively low number of cases in the eGFR<30 subgroup.

Although a host of additional risk factors for PC-AKI apart from decreased renal function have been proposed they have not been sufficiently confirmed on the basis of randomized controlled trials [6, 7]. Accordingly, in this study we were not able to identify an additional patient related risk factor with a significant effect on the occurrence of PC-AKI.

Furthermore there were no significant differences in the rates of PC-AKI between inpatients and outpatients (including day-hospital patients) which is in contradiction to several previous reports [11, 38–40]. Correspondingly, no significant difference between elective and emergency patients could be shown. To what extent our CT-protocol was responsible for the overall incidence of CI-AKI is difficult to attest due to the study design using a single contrast agent (iso-osmolar) at standardized concentration and volume. Our routine CT-examination protocol in cancer patients was developed in accordance with current expert opinions and guidelines for prevention of contrast-induced nephropathy recommending the use of IOCM along with a reduction in CM volume and/or concentration [9, 41, 42]. Combinations of low-kV and low-dose CM have been used successfully in the near past and demonstrated image quality comparable to high-dose protocols [43, 44].

Our study has some limitations. First it is retrospective in character. Second, there is no comparison with either LOCM or different contrast agent volumes or concentrations. Third, not all potential coexisting pathologies (e.g. dehydration) could be excluded as direct causes for AKI due to the retrospective character of our data analysis. Fourth, there was no comparison with controls in the same clinical setting not exposed to iodine contrast agent. Lastly, all patients in the present study received preventive hydration as a sole measure to reduce the incidence of PC-AKI according to the current standards, since the value of using compounds with antioxidant properties other than sodium bicarbonate such as N-acetylcysteine; statins; ascorbic acid; etc., remains controversial [45]. More precisely, most patients received preventive oral hydration before and after CECT, whereas a small number received IV hydration with 500 ml saline 0.9%. However, oral hydration as the sole means of prevention is not recommended and current ESUR CMSC guidelines recommend IV hydration with either 3 ml/kg/h bicarbonate 1.4% for 1 h before CECT or 1 ml/kg/h saline 0.9% for 3–4 h before and 4–6 h after CECT [26].

In conclusion, we found relatively low incidences of PC-AKI (6.3%) and presumed CI-AKI (3.8%). In addition there was no evidence of the baseline eGFR having an effect on the probability of the occurrence of PC-AKI. Hence, the use of a reduced-dose of the iso-osmolar contrast medium iodixanol seems to be safe in high-risk oncology patients with impaired renal function.

## Supporting information

S1 File Complete dataset. (XLSX)

## References

[1] Kooiman J, Pasha SM, Zondag W, Sijpkens YW, van der Molen AJ, Huisman MV, et al Meta-analysis: serum creatinine changes following contrast enhanced CT imaging. Eur J Radiol. 2012;81(10):2554–61. 10.1016/j.ejrad.2011.11.020 22177326

[2] McCullough PA, Sandberg KR. Epidemiology of contrast-induced nephropathy. Rev Cardiovasc Med. 2003;4 Suppl 5:S3–9. 14668704

[3] Ozkok S, Ozkok A. Contrast-induced acute kidney injury: A review of practical points. World J Nephrol. 2017;6(3):86–99. 10.5527/wjn.v6.i3.86 28540198 PMC5424439

[4] Rao QA, Newhouse JH. Risk of nephropathy after intravenous administration of contrast material: a critical literature analysis. Radiology. 2006;239(2):392–7. 10.1148/radiol.2392050413 16543592

[5] Mehran R, Nikolsky E. Contrast-induced nephropathy: definition, epidemiology, and patients at risk. Kidney Int Suppl. 2006(100):S11–5. 10.1038/sj.ki.5000368 16612394

[6] Media ACoDaC. ACR Manual On Contrast Media Version 10.3. 2018.

[7] van der Molen AJ, Reimer P, Dekkers IA, Bongartz G, Bellin MF, Bertolotto M, et al Post-contrast acute kidney injury—Part 1: Definition, clinical features, incidence, role of contrast medium and risk factors: Recommendations for updated ESUR Contrast Medium Safety Committee guidelines. Eur Radiol. 2018;28(7):2845–55. 10.1007/s00330-017-5246-5 29426991 PMC5986826

[8] Davenport MS, Khalatbari S, Cohan RH, Dillman JR, Myles JD, Ellis JH. Contrast material-induced nephrotoxicity and intravenous low-osmolality iodinated contrast material: risk stratification by using estimated glomerular filtration rate. Radiology. 2013;268(3):719–28. 10.1148/radiol.13122276 23579046

[9] Owen RJ, Hiremath S, Myers A, Fraser-Hill M, Barrett BJ. Canadian Association of Radiologists consensus guidelines for the prevention of contrast-induced nephropathy: update 2012. Can Assoc Radiol J. 2014;65(2):96–105. 10.1016/j.carj.2012.11.002 24559602

[10] Zhao F, Lei R, Yang SK, Luo M, Cheng W, Xiao YQ, et al Comparative effect of iso-osmolar versus low-osmolar contrast media on the incidence of contrast-induced acute kidney injury in diabetic patients: a systematic review and meta-analysis. Cancer Imaging. 2019;19(1):38 10.1186/s40644-019-0224-6 31215488 PMC6580528

[11] Hou SH, Bushinsky DA, Wish JB, Cohen JJ, Harrington JT. Hospital-acquired renal insufficiency: a prospective study. Am J Med. 1983;74(2):243–8. 10.1016/0002-9343(83)90618-6 6824004

[12] Cigarroa RG, Lange RA, Williams RH, Hillis LD. Dosing of contrast material to prevent contrast nephropathy in patients with renal disease. Am J Med. 1989;86(6 Pt 1):649–52. 2729314 10.1016/0002-9343(89)90437-3

[13] Moos SI, van Vemde DN, Stoker J, Bipat S. Contrast induced nephropathy in patients undergoing intravenous (IV) contrast enhanced computed tomography (CECT) and the relationship with risk factors: a meta-analysis. Eur J Radiol. 2013;82(9):e387–99. 10.1016/j.ejrad.2013.04.029 23711425

[14] McDonald RJ, McDonald JS, Bida JP, Carter RE, Fleming CJ, Misra S, et al Intravenous contrast material-induced nephropathy: causal or coincident phenomenon? Radiology. 2013;267(1):106–18. 10.1148/radiol.12121823 23360742 PMC6940002

[15] McDonald JS, McDonald RJ, Comin J, Williamson EE, Katzberg RW, Murad MH, et al Frequency of acute kidney injury following intravenous contrast medium administration: a systematic review and meta-analysis. Radiology. 2013;267(1):119–28. 10.1148/radiol.12121460 23319662

[16] Christiansen CF, Johansen MB, Langeberg WJ, Fryzek JP, Sorensen HT. Incidence of acute kidney injury in cancer patients: a Danish population-based cohort study. Eur J Intern Med. 2011;22(4):399–406. 10.1016/j.ejim.2011.05.005 21767759

[17] Launay-Vacher V, Oudard S, Janus N, Gligorov J, Pourrat X, Rixe O, et al Prevalence of Renal Insufficiency in cancer patients and implications for anticancer drug management: the renal insufficiency and anticancer medications (IRMA) study. Cancer. 2007;110(6):1376–84. 10.1002/cncr.22904 17634949

[18] Ng CS, Kalva SP, Gunnarsson C, Ryan MP, Baker ER, Mehta RL. Risk of renal events following intravenous iodinated contrast material administration among inpatients admitted with cancer a retrospective hospital claims analysis. Cancer Imaging. 2018;18(1):30 10.1186/s40644-018-0159-3 30143056 PMC6109283

[19] Perazella M. A. Onco-Nephrology. American Society of Nephrology Online Curriculum. 2016 [Cited 2020 May 10]. Available from: https://www.asn-online.org/api/download/?file=/education/distancelearning/curricula/onco/OncoNephrologyCurriculum.pdf.

[20] Cicin I, Erdogan B, Gulsen E, Uzunoglu S, Sut N, Turkmen E, et al Incidence of contrast-induced nephropathy in hospitalised patients with cancer. Eur Radiol. 2014;24(1):184–90. 10.1007/s00330-013-2996-6 24220752

[21] Cheruvu B, Henning K, Mulligan J, Klippenstein D, Lawrence D, Gurtoo L, et al Iodixanol: risk of subsequent contrast nephropathy in cancer patients with underlying renal insufficiency undergoing diagnostic computed tomography examinations. J Comput Assist Tomogr. 2007;31(4):493–8. 10.1097/rct.0b013e31802e29d9 17882021

[22] McCullough PA, Wolyn R, Rocher LL, Levin RN, O'Neill WW. Acute renal failure after coronary intervention: incidence, risk factors, and relationship to mortality. Am J Med. 1997;103(5):368–75. 10.1016/s0002-9343(97)00150-2 9375704

[23] Salahudeen AK, Doshi SM, Pawar T, Nowshad G, Lahoti A, Shah P. Incidence rate, clinical correlates, and outcomes of AKI in patients admitted to a comprehensive cancer center. Clin J Am Soc Nephrol. 2013;8(3):347–54. 10.2215/CJN.03530412 23243268 PMC3586962

[24] McDonald RJ, McDonald JS, Newhouse JH, Davenport MS. Controversies in Contrast Material-induced Acute Kidney Injury: Closing in on the Truth? Radiology. 2015;277(3):627–32. 10.1148/radiol.2015151486 26599922

[25] Perazella MA. Pharmacology behind Common Drug Nephrotoxicities. Clin J Am Soc Nephrol. 2018;13(12):1897–908. 10.2215/CJN.00150118 29622670 PMC6302342

[26] van der Molen AJ, Reimer P, Dekkers IA, Bongartz G, Bellin MF, Bertolotto M, et al Post-contrast acute kidney injury. Part 2: risk stratification, role of hydration and other prophylactic measures, patients taking metformin and chronic dialysis patients: Recommendations for updated ESUR Contrast Medium Safety Committee guidelines. Eur Radiol. 2018;28(7):2856–69. 10.1007/s00330-017-5247-4 29417249 PMC5986837

[27] KDIGO. KDIGO Clinical Practice Guideline for Acute Kidney Injury. Kidney International Supplements. 2012;2(1):1–138.

[28] Mehta RL, Kellum JA, Shah SV, Molitoris BA, Ronco C, Warnock DG, et al Acute Kidney Injury Network: report of an initiative to improve outcomes in acute kidney injury. Crit Care. 2007;11(2):R31 10.1186/cc5713 17331245 PMC2206446

[29] McDonald JS, McDonald RJ, Williamson EE, Kallmes DF. Is Intravenous Administration of Iodixanol Associated with Increased Risk of Acute Kidney Injury, Dialysis, or Mortality? A Propensity Score-adjusted Study. Radiology. 2017;285(2):414–24. 10.1148/radiol.2017161573 28708022

[30] Cohen EP, Krzesinski JM, Launay-Vacher V, Sprangers B. Onco-nephrology: Core Curriculum 2015. Am J Kidney Dis. 2015;66(5):869–83. 10.1053/j.ajkd.2015.04.042 26060184 PMC5479309

[31] Katzberg RW, Newhouse JH. Intravenous contrast medium-induced nephrotoxicity: is the medical risk really as great as we have come to believe? Radiology. 2010;256(1):21–8. 10.1148/radiol.10092000 20574082

[32] McDonald JS, McDonald RJ, Williamson EE, Kallmes DF, Kashani K. Post-contrast acute kidney injury in intensive care unit patients: a propensity score-adjusted study. Intensive Care Med. 2017;43(6):774–84. 10.1007/s00134-017-4699-y 28213620

[33] Mitchell AM, Jones AE, Tumlin JA, Kline JA. Incidence of contrast-induced nephropathy after contrast-enhanced computed tomography in the outpatient setting. Clin J Am Soc Nephrol. 2010;5(1):4–9. 10.2215/CJN.05200709 19965528 PMC2801649

[34] Aspelin P, Aubry P, Fransson SG, Strasser R, Willenbrock R, Berg KJ, et al Nephrotoxic effects in high-risk patients undergoing angiography. N Engl J Med. 2003;348(6):491–9. 10.1056/NEJMoa021833 12571256

[35] Qian G, Yang YQ, Dong W, Cao F, Chen YD. Comparison of Iodixanol and Iopromide in Patients With Renal Insufficiency and Congestive Heart Failure Undergoing Coronary Angiography by Hemodynamic Monitoring. Angiology. 2017;68(10):907–13. 10.1177/0003319717701868 28401790

[36] Song T, Song M, Ge Z, Li Y, Shi P, Sun M. Comparison of the nephrotoxic effects of iodixanol versus iohexol in patients with chronic heart failure undergoing coronary angiography or angioplasty. J Interv Cardiol. 2017;30(3):281–5. 10.1111/joic.12381 28421628

[37] Terrenato I, Sperati F, Musicco F, Pozzi AF, di Turi A, Caterino M, et al Iodixanol versus iopromide in cancer patients: Evidence from a randomized clinical trial. J Cell Physiol. 2018;233(3):2572–80. 10.1002/jcp.26132 28777459

[38] Hipp A, Desai S, Lopez C, Sinert R. The incidence of contrast-induced nephropathy in trauma patients. Eur J Emerg Med. 2008;15(3):134–9. 10.1097/MEJ.0b013e328270367d 18460952

[39] Mitchell AM, Kline JA. Contrast nephropathy following computed tomography angiography of the chest for pulmonary embolism in the emergency department. J Thromb Haemost. 2007;5(1):50–4. 10.1111/j.1538-7836.2006.02251.x 17026644

[40] Nash K, Hafeez A, Hou S. Hospital-acquired renal insufficiency. Am J Kidney Dis. 2002;39(5):930–6. 10.1053/ajkd.2002.32766 11979336

[41] Del Mastro L, Laghi A, Ronco C. Methods to Address Computed Tomography-Related Risk Factors in Oncology Patients: An Expert Opinion Based on Current Evidence. Blood Purificat. 2018;46(1):56–69.

[42] Heinrich MC, Haberle L, Muller V, Bautz W, Uder M. Nephrotoxicity of Iso-osmolar Iodixanol Compared with Nonionic Low-osmolar Contrast Media: Meta-analysis of Randomized Controlled Trials. Radiology. 2009;250(1):68–86. 10.1148/radiol.2501080833 19092091

[43] Annoni AD, Mancini ME, Andreini D, Formenti A, Mushtaq S, Nobili E, et al Overall evaluability of low dose protocol for computed tomography angiography of thoracic aorta using 80kV and iterative reconstruction algorithm using different concentration contrast media. J Med Imag Radiat On. 2017;61(5):614–21. 10.1111/1754-9485.12608 28345174

[44] Benz DC, Grani C, Hirt B, Moch BH, Mikulicic F, Vontobel J, et al A low-dose and an ultra-low-dose contrast agent protocol for coronary CT angiography in a clinical setting: quantitative and qualitative comparison to a standard dose protocol. Brit J Radiol. 2017;90(1074). 10.1259/bjr.20160933 PMC5602183 28406318

[45] Mamoulakis C, Tsarouhas K, Fragkiadoulaki I, Heretis I, Wilks MF, Spandidos DA, et al Contrast-induced nephropathy: Basic concepts, pathophysiological implications and prevention strategies. Pharmacol Ther. 2017;180:99–112. 10.1016/j.pharmthera.2017.06.009 28642116

